# Geometric Constraints of Visual Space

**DOI:** 10.1177/20416695211055212

**Published:** 2021-11-29

**Authors:** Casper J. Erkelens

**Affiliations:** Experimental Psychology, Helmholtz Institute, 120705 Utrecht University, Utrecht, the Netherlands

**Keywords:** visual space, physical space, perspective-space model

## Abstract

Perspective space has been introduced as a computational model of visual space. The model is based on geometric features of visual space. The model has proven to describe a range of phenomena related to the visual perception of distance and size. Until now, the model lacks a mathematical description that holds for complete 3D space. Starting from a previously derived equation for perceived distance in the viewing direction, the suitability of various functions is analyzed. Functions must fulfill the requirement that straight lines, oriented in whatever direction in physical space, transfer to straight lines in visual space. A second requirement is that parallel lines oriented in depth in physical space, converge to a finite vanishing point in visual space. A rational function for perceived distance, compatible with the perspective-space model of visual space, satisfies the requirements. The function is unique. Analysis of alternative functions shows there is little tolerance for deviations. Conservation of the straightness of lines constrains visual space to having a single geometry. Visual space is described by an analytical function having one free parameter, that is, the distance of the vanishing point.

## Introduction

Visual space is the expanse within which we, that is, human beings, and most animals, perceive objects through vision. In humans, visual space differs from physical space at the scale relevant to vision, especially at long viewing distances. The geometry of visual space has been investigated in numerous studies. Results depended heavily on methods, conditions, and instructions. Consequently, a multitude of ideas and models have been proposed in the literature. In a comprehensive review, Wagner (2012) came to the rather daunting conclusion, namely, that we should see visual space as a family of spaces whose individual geometries differ from each other depending on experimental conditions and mental shifts in the meaning of size and distance. Nevertheless, perspective space has been recently introduced as an appropriate model of visual space (Erkelens, 2015a). The geometry of the model is simple and describes experimental results, ranging from the parallel alleys of Hillebrand (1902) and Blumenfeld (1913) to violation of parallelism (Cuijpers et al., 2000), as well as preservation of collinearity (Cuijpers et al., 2002), as well as judgments of angles between rails and bars oriented in depth (Erkelens, 2015b, 2015c). Furthermore, the geometry of perspective space predicted a mathematical relationship between perceived distance $z_{v}$ and physical distance $z_{p}$ in the viewing direction (Erkelens, 2017). The relationship between $z_{v}$ and $z_{p}$ reads

(1) $$z_{v}=\frac{vd\times z_{p}}{vd+z_{p}},$$

where vd is the finite vanishing distance.

The equation, previously derived by Gilinsky (1951) from a model of binocular visual space (Luneburg, 1950), appeared to describe various experimental results of distance judgments equally well as models proposed for specific tasks (Baird & Wagner, 1991; Foley et al., 2004; Gilinsky, 1951; Li & Durgin, 2012; Ooi & He, 2007; Wu et al. 2004). Although perspective space is a promising model of visual space, it fails a complete, mathematical description. The goal of the present study is to extend the mathematical expression for perceived distance in the viewing direction to an analytical model that holds for all visual directions.

**Figure 1.**
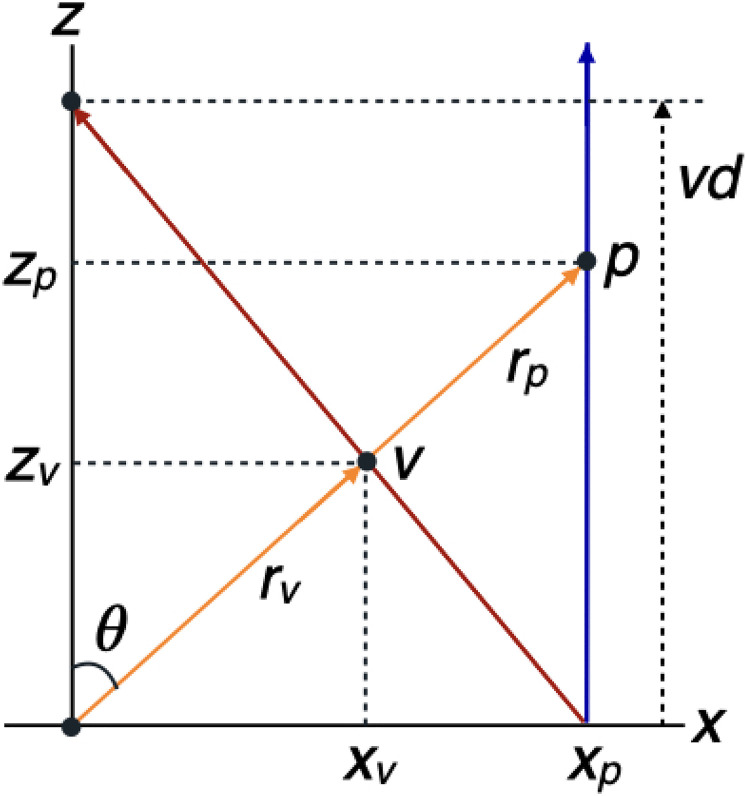
Geometry of perspective space in the transverse plane at eye level. The origin of the Cartesian coordinate system (x,z) coincides with the viewpoint of the observer. The viewing direction is along the z -axis. The line piece starting at $x_{p}$ in the z -direction in physical space (blue) transforms to the line piece directed to the vanishing point at distance vd in perspective space (red). Point *v* represents the physical point *p* in perspective space. Points *p* and *v* are described by their visual direction having an angle θ relative to the z -direction (orange) and distances $r_{p}$ and $r_{v}$ to the viewpoint, in a polar coordinate system.

Until now, perspective space has been described in the form of a geometric construction (Figure 1). Perspective space is defined by two geometric rules. The first rule is that the visual direction $\theta_{v}$ of a point *v* in perspective space coincides with the egocentric direction $\theta_{p}$ of the corresponding point *p* in physical space: $\theta_{v}=\theta_{p}=\theta$ . The second rule of the model is that each point *v* in perspective space is associated with a point *p* in physical space at the intersection of $p^{\prime }s$ egocentric direction and $p^{\prime }s$ perspective line to the vanishing point at a finite distance vd . The two rules are treated as axioms for the mathematical theory of perspective space. The relationship between distances in the viewing direction described by equation (1) has been derived from just these two axioms in other studies (Erkelens, 2017; Wagner et al., 2018).

## Computation of Line Pieces in Perspective Space

Characteristic for perspective space is the property that straight lines, oriented in any random direction in physical space, transfer to straight lines in perspective space (Erkelens, 2015a). Maintaining the straightness of lines is regarded as an essential requirement for the suitability of a mathematical description of perspective space.

To examine mathematical expressions, line pieces have been computed in the horizontal plane of physical and perspective space. Computations started by defining straight line pieces as $z_{p}=a\;x_{p}+b$ for a range of $x_{p}^{\prime }s$ in the (x,z) -plane of physical space. Straight lines have this simple expression in a Cartesian coordinate system. The two axioms underlying perspective space, however, are rules in terms of direction and distance, which are easier expressed in a polar coordinate system. After the conversion of the points $\left(x_{p},z_{p}\right)$ into polar coordinates $\left(r_{p},\theta_{p}\right)$ , the associated points $\left(r_{v},\theta_{v}\right)$ in perspective space were computed from the relationship between physical and perspective positions:

(2) $$(r_{v},\theta_{v})=\left(\frac{vd\times r_{p}}{vd+r_{p}},\;\theta_{p}\right).$$

**Figure 2.**
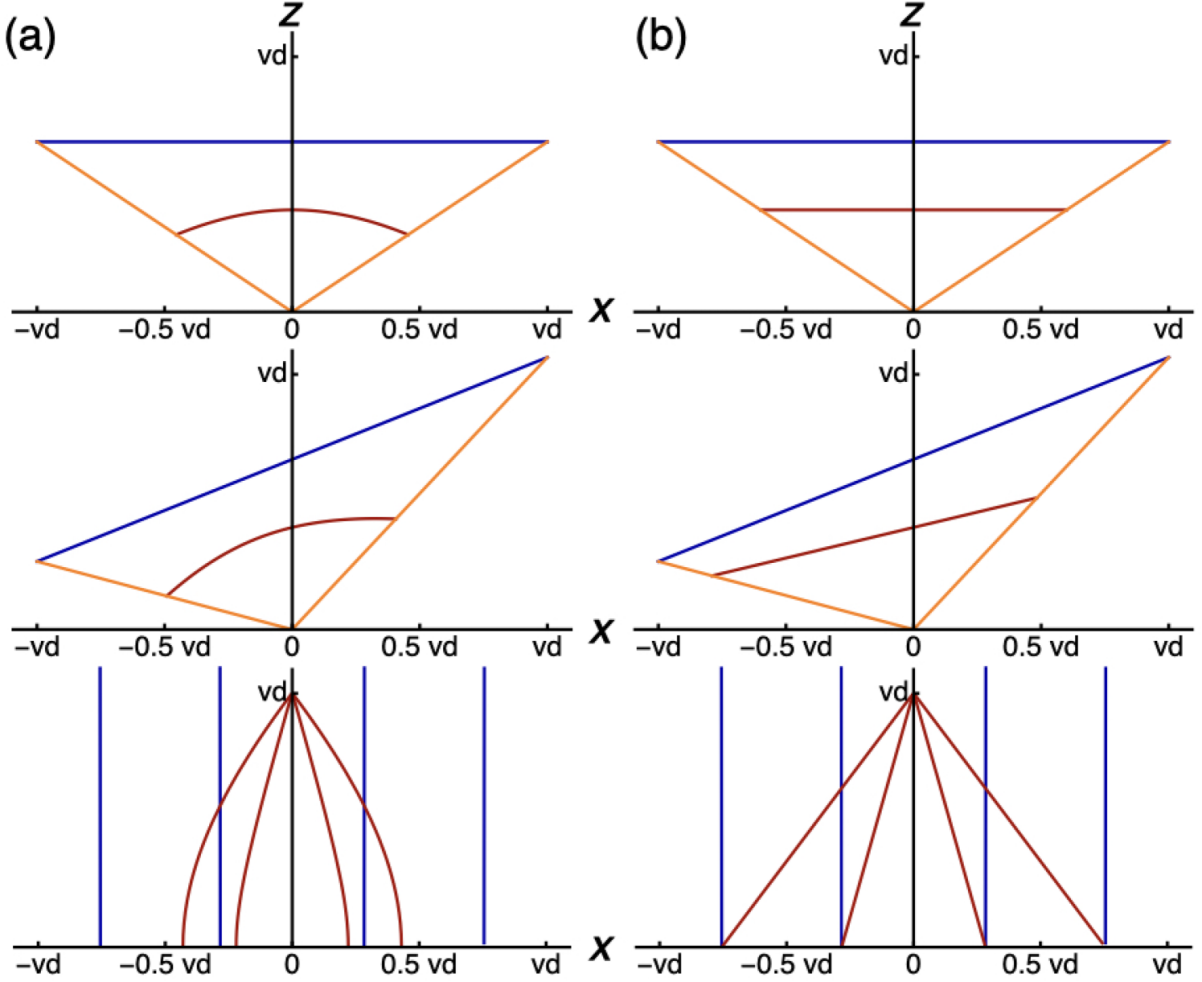
Computed line pieces in the (x,z) -plane. The plots show line pieces between two egocentric directions (orange) in physical (blue) and perspective (red) space. Line pieces in physical space are fronto-parallel (top), slanted (middle), and oriented in depth (bottom). (a) Perspective line pieces were computed from equation (2). (b) Perspective line pieces were computed from equation (3).

This relationship extends the properties of perceived distance in the viewing direction to all visual directions in the horizontal plane. Points $\left(x_{v},z_{v}\right)$ were obtained by converting the points $\left(r_{v},\theta_{v}\right)$ to Cartesian coordinates. The computed line pieces in Figure 2a show that straight line segments in physical space transfer to curved line segments in perspective space, irrespective of the orientation of the straight lines. All lines become curved, except lines along visual directions. The implication of this result is that the used distance relationship is not adequate for perspective space, and thus, not for visual space. The equation for distance $r_{v}$ implies that distance vd is the maximum distance of perspective space in all visual directions. In other words, perspective space is confined to a disc in the transverse plane at eye level. Apparently, this concept is not compatible with the conservation of the straightness of lines. Figure 2a shows perspective line pieces that are concave with respect to the viewpoint, implying that perspective distance is underestimated in directions different from the viewing direction. An alternative model for perspective space may be that the limitation of distances to the vanishing distance vd is restricted to the viewing direction, here taken along the z -axis and perspective distance equals physical distance in the orthogonal directions along the x -axis. In this model, the relationship between physical and perspective positions becomes:

(3) $$(r_{v},\theta_{v})=\left(\frac{vd\times r_{p}}{vd+z_{p}},\;\theta_{p}\right).$$

In this relationship, the term $vd+z_{p}$ replaces the term $vd+r_{p}$ in the denominator of the distance function of equation (2). Figure 2b shows that perspective line segments are straight in all orientations in the revised model. The fact that the sizes along the axes can be expressed in terms of vd , implies that the conservation of straightness of lines holds throughout the horizontal plane for all vanishing distances.

The straightness of perspective line segments has been investigated here in two dimensions. However, placement of the viewpoint at the origin of the plane is the only restriction applied to the computations made for line pieces in two-dimensional planes. Rotation of the plane about the x -axis generalizes the observed properties to all planes of 3D perspective space that include the x -axis. Computations have been extended to 3D space to investigate the straightness of perspective lines, lying in planes that do not include the x -axis. To that end, the 2D polar coordinate system was extended to the 3D spherical coordinate system. In spherical coordinates, the relevant transformation from physical to perspective space is

(4) $$(r_{v},\theta_{v},\varphi_{v})=\left(\frac{vd\times r_{p}}{vd+z_{p}},\;\theta_{p},\varphi_{p}\right).$$

**Figure 3.**
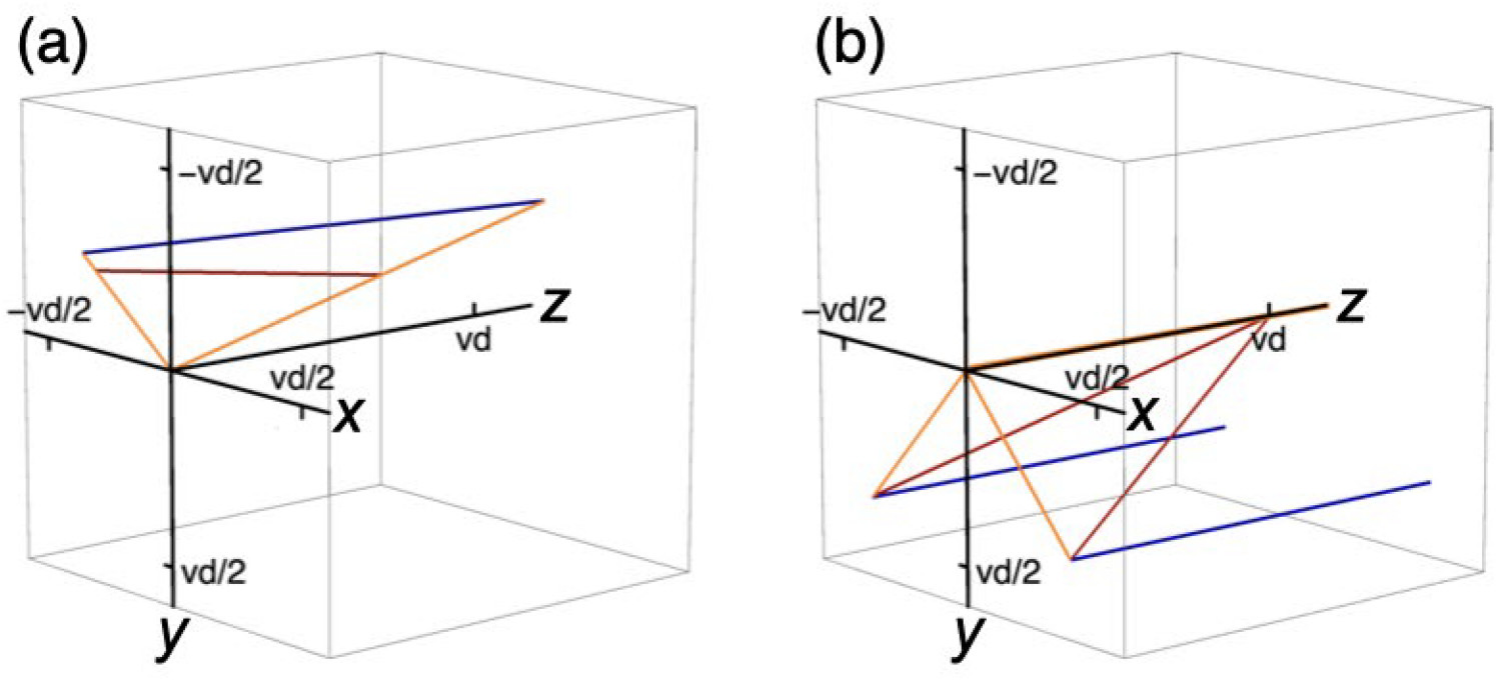
Computed line pieces in 3D space. The plots show line pieces in physical (blue) and perspective (red) space between two egocentric directions (orange). (a) The selected line-pieces do not intersect the x -axis in both physical and perspective space. (b) Two straight physical lines are parallel to each other and to the viewing direction (along the z -axis). The associated perspective lines are straight as well and meet at the vanishing distance vd in the viewing direction.

Figure 3 shows by two examples that straight line segments in 3D physical space induce straight line segments in 3D perspective space. Figure 3a shows line pieces having generic directions and Figure 3b shows the situation of a person looking at the far end of a straight railway track. The tracks meet in a vanishing point at a finite distance vd in perspective space, just as they do in visual space (Erkelens, 2015b).

## Computation of Equidistance Loci

Positions of apparent equal distance have been a subject of considerable interest in both monocular and binocular vision (Howard & Rogers, 2012). Together with such positions, called the locus of equidistant points, constitute a circle in the transverse plane at eye level of physical space. The equidistance locus in the same plane of perspective space is given by the equation:

(5) $$r_{v}=\frac{vd\times r_{p}}{vd+z_{p}}=\mathrm{constant}.$$

**Figure 4.**
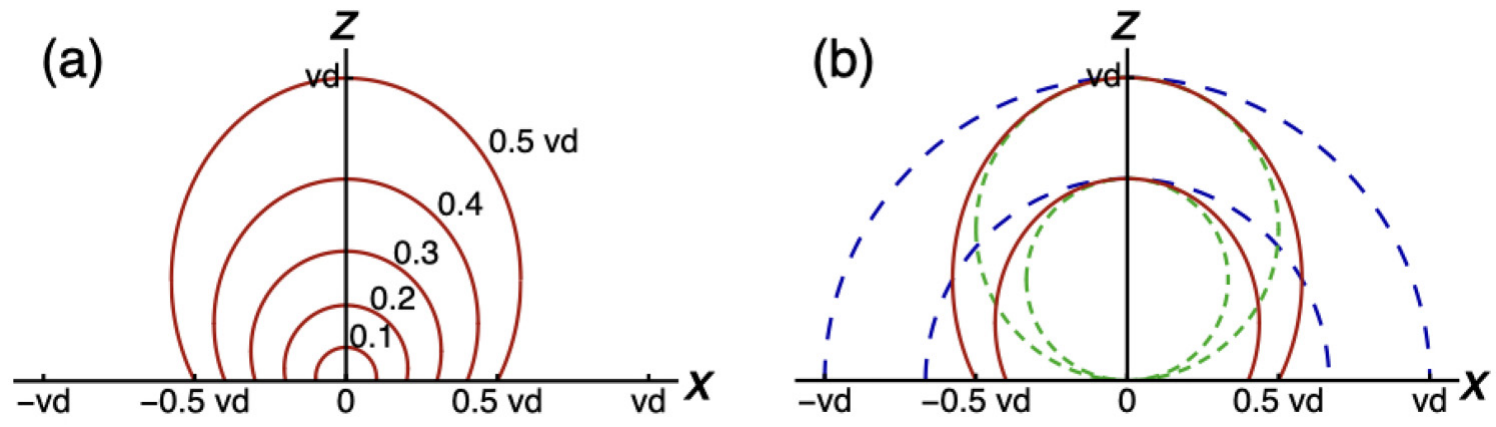
Computed loci of egocentric equidistance. (a) The loci fulfill the constraint $r_{v}$ is constant. The constants are from 0.1vd to 0.5vd . (b) Loci of perspective equidistance (red) are drawn together with loci of physical equidistance (blue) and Vieth-Müller circles (green).

Figure 4a shows computed loci of which individual points are equidistant from the viewpoint in perspective space. On the x -axis, that is, orthogonal to the viewing direction, $x_{v}=x_{p}$ . Thus, perceived distance equals physical distance along the x -axis. The relationship is different along the z -axis, that is, in the viewing direction. For instance, take the case $r_{v}=0.5\;vd$ . From equation (5) it follows that $z_{p}=vd$ . Thus, for this case, the locus of equidistance lies twice as far from the viewpoint in the viewing direction as in the orthogonal directions. The observation that equidistantly perceived stimuli are set at the farthest physical distance in the viewing direction, implies that visual space has been compressed maximally in the viewing direction. The equidistance curves show that the degree of compression gradually decreases with increasing eccentricity until it becomes zero in the orthogonal directions.

In Figure 4b, two equidistance loci in perspective space are drawn together with similar loci in physical space and loci lying on Vieth-Müller circles. As was remarked, the loci in physical space are circles about the viewpoint. Vieth-Müller circles, or horizontal horopters, are loci in the horizontal plane of which points project to identical retinal locations of the two eyes. The horopters include the binocular fixation point and the nodal points of the eyes. Horopters are relevant for a discussion of equidistance loci because it has been proposed in the literature that objects projecting to corresponding retinal points appear equidistant to observers (Howard & Rogers, 2012). Figure 4b shows that equidistance loci in perspective space lie in between horopters and equidistant loci in physical space. This observation is of interest in relation to the results of equidistance judgments reported in the literature and will be discussed in the Discussion section.

## Computation of Line Pieces Based on Another Model of Depth Perception

Until now, computations have been made for perspective space as a model of visual space. Good results were obtained if the perceived distance was described by the rational function $r_{v}=vd\;r_{p}/(vd+z_{p})$ . Perspective space represents a class of models that share the assumption of a finite visual space. There are also models that assume an infinite visual space. Those models may be represented by a model, in which judged distances are described by power functions of the form $r_{v}=\kappa \;r_{p}^{\gamma }$ , where κ is a scaling factor and γ the exponent (Wagner, 2012).

**Figure 5.**
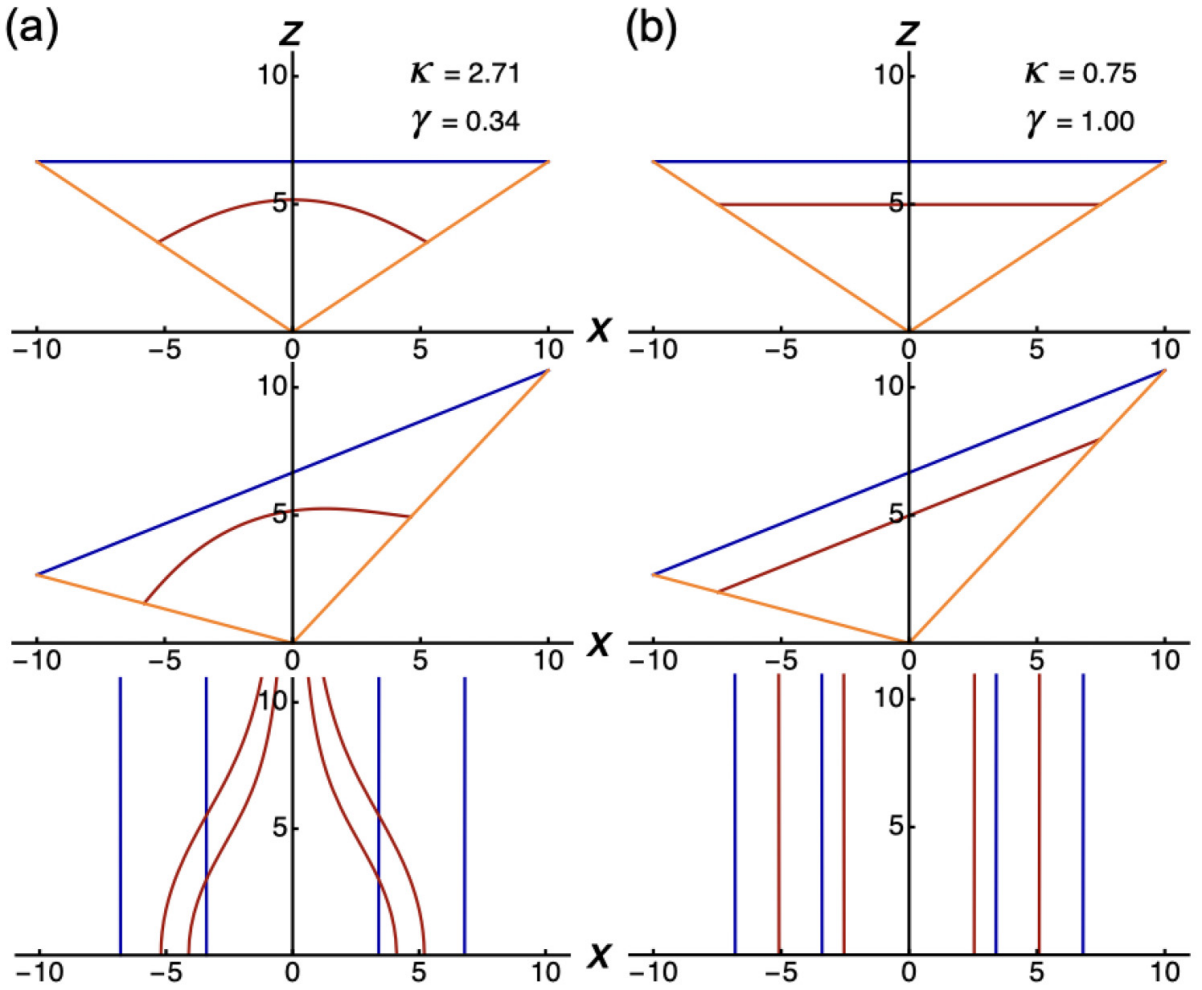
Computed line pieces in the (x,z) -plane. The model of visual space is based on power functions fitted to results of depth judgments. The plots show line pieces between two egocentric directions (orange) in physical (blue) and modeled (red) space. Line pieces in physical space are fronto-parallel (top), slanted (middle), and oriented in depth (bottom). (a) Perspective line pieces are computed for the parameter combination κ=2.71 , γ=0.34 . (b) Parameters are κ=0.75 , γ=1.00 .

Line pieces have been computed for a range of parameter values of κ and γ . Figure 5a shows characteristic line pieces. Line pieces are concave for γ<1 and convex for γ>1 . Figure 5b shows line pieces for the special case of γ=1 . Then, all computed line pieces are straight. However, lines oriented in depth do not converge to a finite vanishing point but are parallel to each other, and the lines in physical space. In this case, modeled space is a linear transformation of physical space.

## Analysis of the Analytical Function for Distance in Perspective Space

The previous paragraph showed that models assuming an infinite space cannot describe visual space because parallel lines oriented in depth in physical space will not appear to converge. Finite vanishing points such as in perspective space are a prerequisite for the description of visual space. Perspective space appears to describe visual space very accurately if its finite distance is defined for the viewing direction. The non-linear relationship between visual and physical distance in the viewing direction is unique because it follows directly from the two geometric axioms. The question arises whether the demand of straightness of lines tolerates any deviation from this relationship. To address this question, computations were made for slightly different distance relationships.

**Figure 6.**
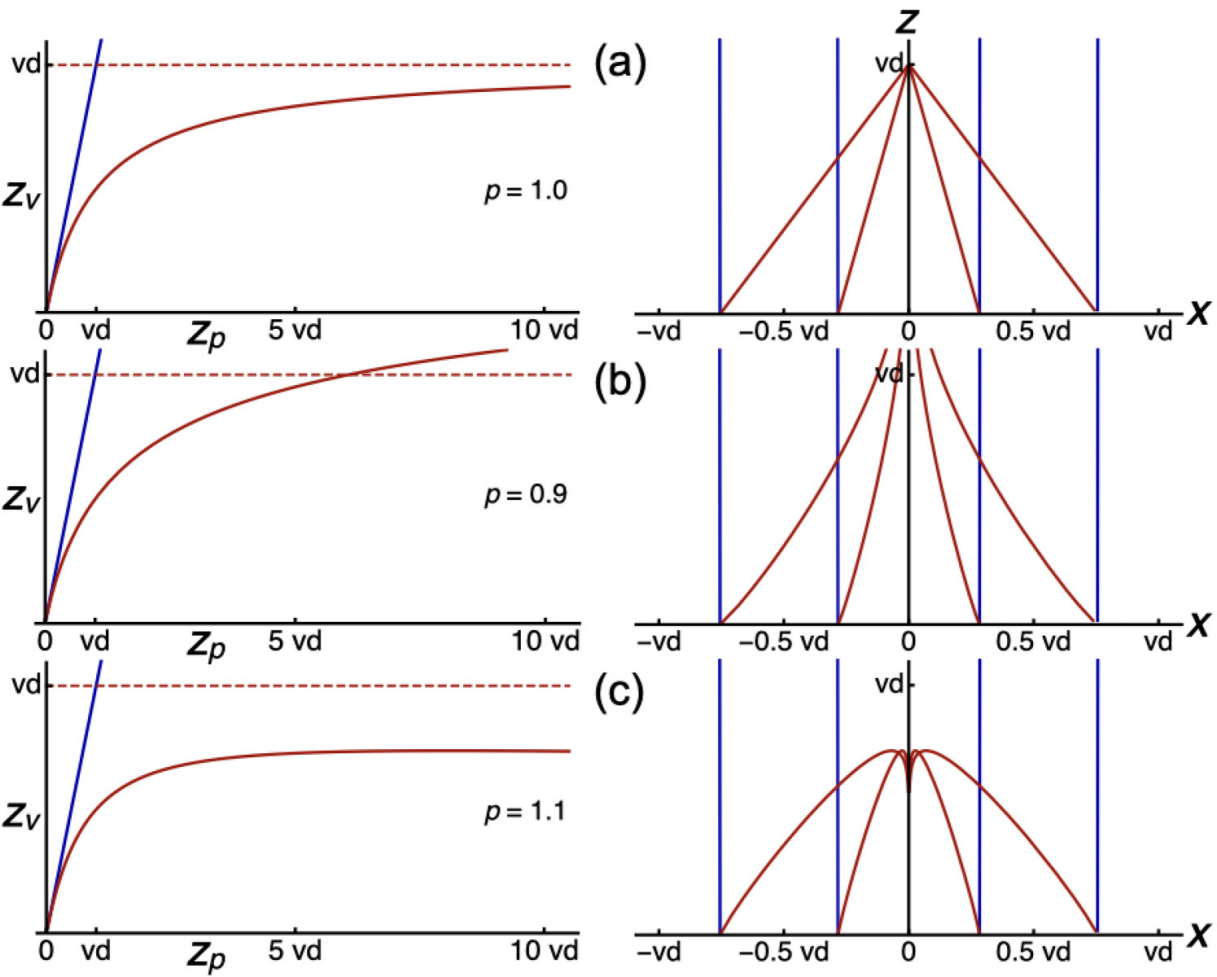
Computed distance relationships and line pieces in the (x,z) -plane. Lines are blue in physical space and red in perspective space. Plots on the left show perceived distance $z_{v}$ as a function of physical distance $z_{p}$ , given by $z_{v}=vd\;z_{p}/(vd+z_{p}^{p})$ , where p=1.0 in (a), p=0.9 in (b), and p=1.1 in (c). Plots on the right show associated line pieces for which perceived distance is given by $r_{v}=vd\;r_{p}/(vd+z_{p}^{p})$ .

Figure 6a shows $z_{v}$ as a function of $z_{p}$ given by $z_{v}=\;vd\;z_{p}\;/\;(vd+z_{p})$ . For distances $z_{p}<<vd$ , it holds that $z_{v}≅z_{p}$ . Thus, perspective distance is very similar to the physical distance at the short-range. Alternative functions for $z_{v}$ must fulfill the same requirement. The distance function was changed by adding an exponent to $z_{p}$ in the denominator: $z_{v}=vd\;z_{p}/(vd+z_{p}^{p})$ , where $z_{p}$ is raised to the power of *p*. For this function at distances $z_{p}<<vd$ , it also holds that $z_{v}≅z_{p}$ . The left plot of Figure 6b shows $z_{v}$ as a function of $z_{p}$ for the value of p=0.9 . A major difference to the $z_{v}$ of Figure 6a is that the vanishing distance is shifted from vd to infinity. The right plot of Figure 6b shows that, already for the modestly different function for $z_{v}$ , the converging lines become really curved. A value of *p* higher than 1, for instance, p=1.1 , results in reduced distances of $z_{v}$ as a function of $z_{p}$ (left plot of Figure 6c). Now, the vanishing distance is shifted from vd to 0. As a result, converging lines to the vanishing point bend backwards (right plot of Figure 6c). The very different behaviors of $z_{v}$ for slightly different values of *p*, show that $z_{v}$ is a highly non-linear function of *p*. The effect of small variations of *p* indicates that there is hardly any room for alternative analytical functions describing the geometry of visual space.

## Discussion

The computations established two properties of visual space: (1) visual space is finite, and (2) visual space has a fixed geometry in people with normal vision. Computations showed that visual space must be finite because parallel lines oriented in depth remain parallel in infinite visual spaces (Figure 4b). A finite visual space implies that all models and descriptions based on power functions must be dismissed (see chapter 5 of Wagner (2012) for a meta-analysis on data from a long list of studies). Visual space appears to have a very specific geometry. It is the only geometry warranting that, for instance, straight railway tracks, oriented in depth, are perceived as straight, converging tracks (Erkelens, 2015b).

The function describing the geometry of perspective space was found by combining experimental judgments with everyday observation. The experimental judgments are the estimated distances of objects, such as have been measured in many experiments. The everyday observation is that straight lines in physical space are also straight in visual space. Distances in the perspective-space model are compatible with distances produced by several other models, proposed to describe the results of different types of distance judgments (Erkelens, 2017). Most of the models are confined to describing distance and size judgments for objects placed in the viewing direction of observers. They are not defined for other directions of visual space. The perspective-space model was construed to describe visual space in all directions. Until now, however, it gave just a mathematical expression for distances and sizes in the viewing direction. The current analysis extends the mathematically formulated geometry to the entire 3D space. Establishing that straight lines in physical space are also straight in visual space, seems obvious if we look at the shapes of objects in our direct environment. For example, straight lines are abundantly present in buildings, rooms, windows, tiles, and many other objects. Would designers, constructors, and builders have taken the effort to design and make objects having straight edges if these would be perceived as curved? For instance, makers of camera lenses go to great lengths to minimize barrel and pin-cushion distortions as much as possible. Still, in the literature of space perception, the longstanding conviction is that visual space is curved. Ideas of a curved visual space are mainly based on indirect measurements of positions and orientations of small, isolated objects. Famous are the parallel and distance alleys in depth, initially measured by Hillebrand (1902) and Blumenfeld (1913). The alleys led to the concept of curved visual space (Luneburg, 1947, 1950). The alleys are described by the perspective-space model, if one accepts that parallel alleys reflect a special condition in visual space and distance alleys a special condition in physical space (Erkelens, 2015a, 2017). Later, experiments of Cuijpers et al. (2000) showed that parallelism in physical space is violated in visual space, also suggesting curvedness. However, the perspective model of a flat visual space, in which geodesics are straight lines, appeared to describe these results too (Erkelens, 2015a). A direct demonstration of curved lines that appear straight has been reported by von Helmholtz (1910/1925/2000). He observed that a 90-degree-wide pin-cushion pattern was seen as a squared checkerboard if it was fixated monocularly from a distance of 20 cm. The effect was probably dominated by non-perceived distortions in the far periphery because the effect was greatly reduced during free viewing (Oomes et al., 2009). During fixation from a distance of 20 cm, the central pattern is seen sharp, whereas the peripheral pattern becomes progressively blurred towards the edges. An alternative interpretation of Helmholtz's observation is that the flat pin-cushion pattern is perceived as a concave sphere, of which all lines bend towards the viewer. In fact, such observations were made by divers wearing facemasks while viewing a planar structure underwater (Vernoy & Luria, 1977). The alternative interpretation of Helmholtz’s observation implies that curved lines in physical space appear as curved in visual space. And thus, the demonstration by Helmholtz does not contradict the contention that straight lines in physical space are also straight in visual space.

A remarkable result of the computations is the fact that equidistance loci in perspective space are lying in between circles about the viewpoint and Vieth-Müller circles (Figure 4). Foley (1966) measured the locus of perceived equidistance in the eye-level plane at several distances from the observer. This locus was found to be concave with a curvature intermediate between the physically equidistant circle and that of the corresponding Vieth-Müller circle. Ebenholtz and Ebenholtz (2003) used another method and came to a similar conclusion during binocular and monocular viewing of the stimuli in further darkness. These results are in line with earlier measurements of the empirical longitudinal horopter by Hering (1864) and Hillebrand (1893). The measurements showed a consistent deviation from the geometric Vieth-Müller circle, whether the horopter was measured in terms of equal visual direction or by the more perceptual criteria of the range of fusion or equal perceived distance (Tyler, 1991). The measured points were located outside of the Vieth-Müller circle. The deviation between the empirical and theoretical horopter, known as the Hering-Hillebrand deviation, was explained by a supposed asymmetry between the nasal and temporal retinae (Ogle, 1962). The measured loci of equidistance discussed here, are in close agreement with the computed equidistance loci of perspective space. This observation suggests that the empirical horopter is better explained by the geometry of visual space than by the theoretical horopter.

The analytical model is not just the appropriate description of visual space, it is also a simple description. Expressed in vector notation, the description is even simpler. In vector notation, the relationship between positions in visual and physical space is given by

(6) $$\vec{v}=\left(\frac{vd}{vd+\vec{\;p}\;.\;\hat{z}}\right)\vec{\;p}\;,$$

where $\vec{v}$ and $\vec{\;p}$ are the positions in visual and physical space, relative to the viewpoint, and $\hat{z}$ is the unit vector in the viewing direction. Equation (6) is equivalent to equation (1) in the viewing direction, to equation (3) in the horizontal plane, and to equation (4) in 3D space. The scalar product $\vec{\;p}\;.\;\hat{z}$ is at maximum in the viewing direction and decreases with an increasing angle between $\vec{\;p}\;$ and $\hat{z}$ . Consequently, perceived distance, the distance of $\vec{v}$ from the viewpoint, is at minimum in the viewing direction and becomes less compressed at increasing eccentricities. This property of perceived distance has been demonstrated by the experimental equidistance loci.

The relationship is valid throughout 3D space and relates positions in visual space one-to-one to positions in physical space. Analysis of alternative functions showed that the closed-form expression for $\vec{v}$ is unique. There is not another rational function that leaves the straightness of lines intact. This means that the relationship is implicitly embedded in the visual systems of all individuals, who perceive straight lines and contours straight. The parameter vd is the only quantity that may vary in individual people, just as vd varies in different tasks and conditions (Erkelens, 2015a, 2015b, 2015c, 2017). Variation in the magnitude of vd is associated with variation in the perceived depth of objects. Straight lines in any direction, however, remain straight (Erkelens, 2015a). Growing of vd may be experienced during prolonged (monocular) viewing of a painting that depicts a scene containing much depth.

Perspective space is not a neurobiological model of visual space. It does not explain or even suggest how visual space emerges from retinal images and neural processes (Erkelens, 2017). Neural implementation of the function for perceived distance is highly unlikely because it would require knowledge of the physical distance of objects. The concept of a visual space different from physical space would be redundant if the brain would have accurate knowledge of physical space. This raises the question: How does the brain create and maintain a particular geometry of visual space? The strong relationship between the geometry of visual space and the straightness of lines is suggestive of a neural mechanism. A consequence of the unique relationship between physical and visual distance is that straight lines and contours would look curved with any other geometry of visual space. With another geometry, perceived curvatures of lines and contours of an object would also change in shape, if the object would move, not only in depth but also in other directions. In other words, conservation of straightness dictates the geometry of visual space. This opens the possibility that the geometry of visual space results from a process, serving to keep the perceived shape of objects constant in a dynamic world. Researchers on the statistics of natural scenes have proposed a conceptual framework, in which the statistical properties of the visual environment tune and adapt visual perception (Geisler, 2008; Yang & Purves, 2003). Within this concept, the strong constraint on the geometry of visual space, demanded by the conservation of straightness of lines, may be achieved by a mechanism, dedicated to preserving the curvature of lines and contours over space and time by adjusting their perceived distance. Studies of after-effects have established long ago that visual perception adapts to curvature (Carlson, 1963; Coltheart, 1971; Gibson, 1933). Prolonged inspection of a curved line makes a straight line appear to be curved in the opposite direction. Extensive work on adaptation to prismatic and refractive distortions suggested an internal readjustment tied to the egocentric coordinate system (Harris, 1965; Held & Freedman, 1963; Kohler, 1962). Until now, measurements have been confined to perceived shape. The current study suggests that adaptation to prismatic and refractive distortions may also affect perceived distance.

## References

[bibr1-20416695211055212] Baird, J. C., & Wagner, M. (1991). Transformation theory of size judgment. Journal of Experimental Psychology: Human Perception and Performance, 17(3), 852–864. 10.1037/0096-1523.17.3.852 1834796

[bibr2-20416695211055212] Blumenfeld W . (1913). Untersuchungen über die scheinbare Grösse im Sehraume. Zeitschrift für Psychologie, 65, 241–404.

[bibr3-20416695211055212] Carlson V. R . (1963). The generality of negative aftereffect following adaptation to curvature. Scandinavian Journal of Psychology, 4(1), 129–133. 10.1111/j.1467-9450.1963.tb01317.x

[bibr4-20416695211055212] Coltheart M . (1971). Visual feature-analyzers and after-effects of tilt and curvature. Psychological Review, 78(2), 114–121. 10.1037/h0030639 5547374

[bibr5-20416695211055212] Cuijpers R. H. Kappers A. M. L. Koenderink J. J . (2000). Large systematic deviations in visual parallelism. Perception, 29(12), 1467–1482. 10.1068/p3041 11257970

[bibr6-20416695211055212] Cuijpers R. H. Kappers A. M. L. Koenderink J. J . (2002). Visual perception of collinearity. Perception & Psychophysics, 64(3), 392–404. 10.3758/BF03194712 12049280

[bibr7-20416695211055212] Ebenholtz S. M. Ebenholtz J. M . (2003). Distance perception for points at equiconvergence and equidistance loci. Perception, 32(6), 707–716. 10.1068/p3337 12892431

[bibr8-20416695211055212] Erkelens C. J . (2015a). The perspective structure of visual space. i-Perception, 6(5), 1–13. 10.1177/2041669515613672 27648222 PMC5016827

[bibr9-20416695211055212] Erkelens C. J . (2015b). The extent of visual space inferred from perspective angles. i-Perception, 6(1), 5–14. 10.1068/i0673 26034567 PMC4441024

[bibr10-20416695211055212] Erkelens C. J . (2015c). Perception of perspective angles. i-Perception, 6(3), 1–11. 10.1177/2041669515593022 27433312 PMC4934606

[bibr11-20416695211055212] Erkelens C. J . (2017). Perspective space as a model for distance and size perception. i-Perception, 8(6), 1–20. 10.1177/2041669517735541 PMC5714114 29225765

[bibr37-20416695211055212] Foley, J. M. (1966). Locus of perceived equidistance as a function of viewing distance. *Journal of the Optical Society of America, 56*(6), 822–827. 10.1364/JOSA.56.000822. 5963529

[bibr12-20416695211055212] Foley J. M. Ribeiro-Filho N. P. Da Silva J. A . (2004). Visual perception of extent and the geometry of visual space. Vision Research, 44(2), 147–156. 10.1016/j.visres.2003.09.004 14637364

[bibr13-20416695211055212] Geisler W. S . (2008). Visual perception and statistical properties of natural scenes. Annual Review of Psychology, 59, 167–192. 10.1146/annurev.psych.58.110405.085632 17705683

[bibr14-20416695211055212] Gilinsky A. S . (1951). Perceived size and distance in visual space. Psychological Review, 58(6), 460–482. 10.1037/h0061505 14900306

[bibr15-20416695211055212] Gibson J. J . (1933). Adaptation, after-effect and contrast in the perception of curved lines. Journal of Experimental Psychology, 16(1), 1–31. 10.1037/h0074626

[bibr16-20416695211055212] Harris C. S . (1965). Perceptual adaptation to inverted, reversed, and displaced vision. Psychological Review, 72(6), 419–444. 10.1037/h0022616 5322170

[bibr17-20416695211055212] Held R. Freedman S. J . (1963). Plasticity in human sensorimotor control. Science (New York, NY), 142(3591), 455–462. 10.1126/science.142.3591.455 14064442

[bibr18-20416695211055212] Helmholtz H. von . (1910/1925/2000). The monocular field of vision. In Southall J. P. C. (ed.), Helmholtz’s treatise on physiological optics. Volume 3, Chap. 28, pp. 154–242. Thoemmes Press.

[bibr35-20416695211055212] Hering, E. (1864). *Beiträge zur Physiologie: Vom binocularen Tiefsehen: Kritik einer Abhandlung von Helmholtz über den Horopter*. Verlag von Wilhelm Engelmann.

[bibr36-20416695211055212] Hillebrand F. (1893). Die Stabilität der Raumwerte auf der Netzhaut. *Zeitschrift für Psychologie und Physiologie des Sinnesorgane, 5*, 1–60.

[bibr19-20416695211055212] Hillebrand F . (1902). Theorie der scheinbaren Grösse bei binocularem Sehen. Denkschriften Der Wiener Akademie, Mathematisch-Naturwissenschaftliche Klasse, 72, 255–307.

[bibr20-20416695211055212] Howard I. P. Rogers B. J. (2012). Perceiving in depth: Volume 2: Stereoscopic vision. Oxford University Press.

[bibr21-20416695211055212] Kohler I . (1962). Experiments with goggles. Scientific American, 206(5), 62–73. 10.1038/scientificamerican0562-62 14457820

[bibr22-20416695211055212] Li Z. Durgin F. H . (2012). A comparison of two theories of perceived distance on the ground plane: The angular expansion hypothesis and the intrinsic bias hypothesis. i-Perception, 3(5), 368–383. 10.1068/i0505 22792434 PMC3393602

[bibr23-20416695211055212] Luneburg R. K. (1947). Mathematical analysis of binocular vision. Princeton University Press.

[bibr24-20416695211055212] Luneburg R. K . (1950). The metric of binocular visual space. Journal of Optical Society of America, 40(10), 637–642. 10.1364/JOSA.40.000627

[bibr25-20416695211055212] Ogle K. N . (1962). The visual space sense. Science (New York, NY), 135(3506), 763–771. 10.1126/science.135.3506.763 14481308

[bibr26-20416695211055212] Ooi T. L. He Z. J . (2007). A distance judgment function based on space perception mechanisms: Revisiting Gilinsky’s (1951) equation. Psychological Review, 114(2), 441–454. 10.1037/0033-295X.114.2.441 17500634

[bibr27-20416695211055212] Oomes A. H. Koenderink J. J. van Doorn A. J. de Ridder H . (2009). What are the uncurved lines in our visual field? A fresh look at Helmholtz’s checkerboard. Perception, 38(9), 1234–1294. 10.1068/p6288 19911627

[bibr28-20416695211055212] Tyler C. W. (1991). The horopter and binocular fusion. In Regan D. (ed.), Vision and visual dysfunction, volume 9: Binocular vision (pp. 19–38). The Macmillan Press Ltd.

[bibr29-20416695211055212] Vernoy M. W. Luria S. M . (1977). Perception of, and adaptation to, a three-dimensional curvature distortion. Perception & Psychophysics, 22(3), 245–248. 10.3758/BF03199686

[bibr30-20416695211055212] Wagner M. (2012). The geometries of visual space. Lawrence Erlbaum Associates.

[bibr31-20416695211055212] Wagner M. Hatfield G. Cassese K. Makwinski A. N . (2018). Differentiating between affine and perspective-based models for the geometry of visual space based on judgments of the interior angles of squares. Vision, 2(2), 1–22. 10.3390/vision2020022 PMC6835612 31735886

[bibr32-20416695211055212] Wu B. Ooi T. L. He Z. J . (2004). Perceiving distance accurately by a directional process of integrating ground information. Nature, 428(6978), 73–77. 10.1038/nature02350 14999282

[bibr33-20416695211055212] Yang Z. Purves D . (2003). Image/source statistics of surfaces in natural scenes. Network: Computation of Neural Systems, 14(3), 371–390. 10.1088/0954-898X_14_3_301 12938763

